# The induced motion effect is a high-level visual phenomenon: Psychophysical evidence

**DOI:** 10.1177/20416695221118111

**Published:** 2022-09-07

**Authors:** Michael Falconbridge, Kassandra Hewitt, Julia Haille, David R. Badcock, Mark Edwards

**Affiliations:** 2720 The University of Western Australia, Australia; 2720 The Australian National University, Australia

**Keywords:** higher-order motion, local motion, models, motion, neural mechanisms, optic flow, perception, perceptual organization, scene perception

## Abstract

Induced motion is the illusory motion of a target away from the direction of motion of the unattended background. If it is a result of assigning background motion to self-motion and judging target motion relative to the scene as suggested by the flow parsing hypothesis then the effect must be mediated in higher levels of the visual motion pathway where self-motion is assessed. We provide evidence for a high-level mechanism in two broad ways. Firstly, we show that the effect is insensitive to a set of low-level spatial aspects of the scene, namely, the spatial arrangement, the spatial frequency content and the orientation content of the background relative to the target. Secondly, we show that the effect is the same whether the target and background are composed of the same kind of local elements—one-dimensional (1D) or two-dimensional (2D)—or one is composed of one, and the other composed of the other. The latter finding is significant because 1D and 2D local elements are integrated by two different mechanisms so the induced motion effect is likely to be mediated in a visual motion processing area that follows the two separate integration mechanisms. Area medial superior temporal in monkeys and the equivalent in humans is suggested as a viable site. We present a simple flow-parsing-inspired model and demonstrate a good fit to our data and to data from a previous induced motion study.

## Introduction

Induced motion or the Duncker effect is a compelling visual illusion that has been studied by psychologists for over a century (Carr & Hardy, 1920; Duncker, 1929; Thelin, 1927). Originally the effect referred to the illusory motion of a stationary object when a surrounding or nearby object moved—the sense that the moon is moving when seen near wind-blown clouds being an often-cited example (see Reinhardt-Rutland, 1988 for a review). Now, more generally, it refers to the “repulsion” of target motion away from the direction of the unattended object/s in the scene; a motion vector opposite to that of the unattended object/s is added to the target whether the target moves or not (Bassili & Farber, 1977; Farrell-Whelan et al., 2012; Gogel, 1979; Wallach & Becklen, 1983; Zivotofsky et al., 1995; Zivotofsky, 2004). For example, when a target pattern moves upward against a background pattern that moves to the right, the target will appear to move upward, as expected, but also to the left, away from the background direction of motion.

**Figure 1.**
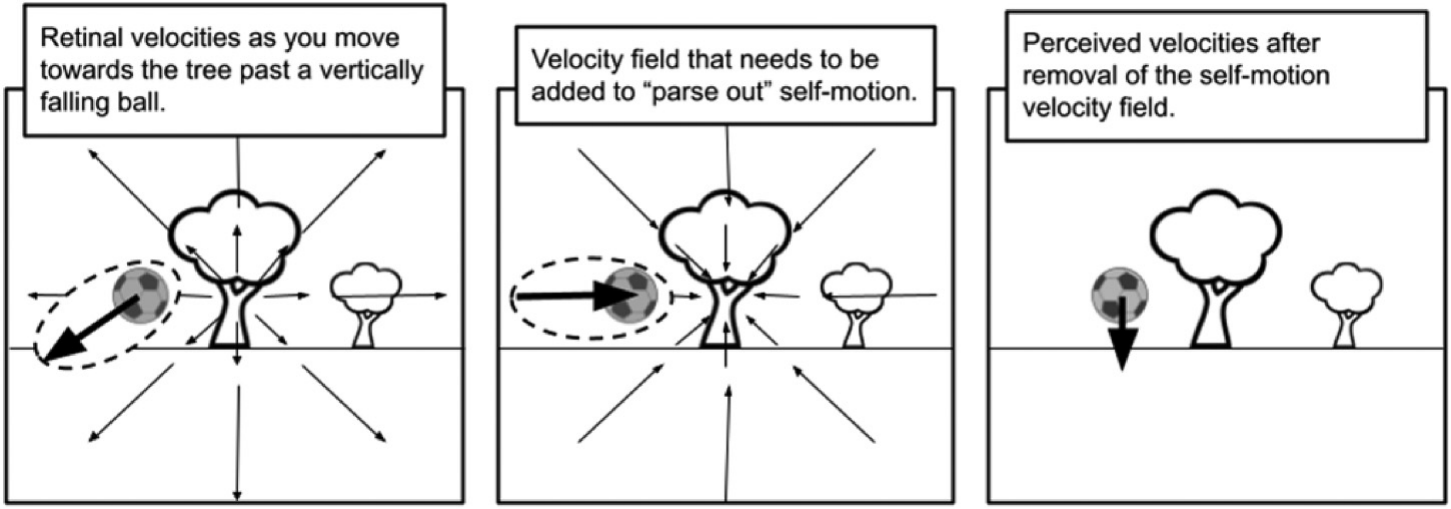
Illustration of the flow parsing hypothesis (Warren & Rushton, 2007). Based on an image in Warren and Rushton (2009). Obtaining the world-centered velocities of the background and object (right panel) from the retinal velocities (left panel) involves subtracting the velocities created by self-motion through the environment, that is, adding the negative of the velocity field created by self-motion (central panel). Note that the oblique retinal motion of the ball is perceived (correctly) as vertical after parsing out self-motion.

During everyday experiences such as catching balls on the run or avoiding obstacles while driving, we accurately calculate target motion in the presence of background motion (arising from self-motion), so why the errors seen in the induced motion illusion? An explanation may lie in a relatively recent proposal to view this two-dimensional (2D) visual phenomenon within a three-dimensional (3D) context. Warren and Rushton (2007) noted that in a real-world, 3D scenario where background motion is due to self-motion, the visual motion caused by self-motion needs to be “parsed out” of the visual scene in order to extract the absolute (world-centered) motion of the target. Parsing out equates to subtracting the background motion in the vicinity of the target (see Figure 1). They suggest that this subtraction may be what causes the repulsion with 2D translating stimuli such as those seen in induced motion experiments (Rushton & Warren, 2005; Warren & Rushton, 2004, 2007, 2008, 2009). Subtracting background motion from target motion effectively adds to the target a motion component in the opposite direction to the background leading to a perceived repulsion away from the background.

Their proposal relies on the unattended motion being treated as a case of optic flow by the visual system. Optic flow is the type of motion that arises in our visual fields as we move through real environments. As optic flow has to be abstracted from low-level motion signals that can be scattered as wide as the entire visual field and because its cause, ego-motion, is associated with other cues such as those from the vestibular system, it is thought to be represented in relatively late stages of the visual processing pathway (Cullen, 2011; Lappe et al., 1999; Perrone, 1992). Mounting evidence suggests it is represented in area medial superior temporal (MST) in the monkey (Duffy & Wurtz, 1991; Gu et al., 2007; McCool & Britten, 2008; Wurtz, 1998) and, equivalently, area hMST in humans (Smith et al., 2006, 2012; Wall & Smith, 2008) at the earliest. This puts the locus of the background/target interaction seen in the induced motion effect at a late stage in the visual motion processing stream.

Our aim was to conduct psychophysical tests of the plausibility of a flow-parsing explanation for the induced motion effect. We looked, specifically, at two requirements if the proposal is to hold. Firstly, the repulsive interaction between target and background needs to occur at a relatively late stage of visual processing, say area MST where optic-flow is processed. Secondly, there is a need to reconcile the proposal with previous research; the proposal demands that the unattended motion in the scene be treated as motion belonging to a stationary background against which an observer is moving but previous psychophysical experiments show induced motion even when the unattended motion belongs to seemingly non-background-like elements such as single dots (e.g., Carr & Hardy, 1920; Duncker, 1929) and other shaped objects (e.g., Farrell-Whelan et al., 2012; Levi & Schor, 1984; Post & Heckmann, 1986; Wallach & Becklen, 1983). If single moving objects work just as well as dispersed motion fields for induced motion then the concept of what constitutes an optic-flow background under the flow-parsing hypothesis needs to be rather broad.

In order to assess whether these two requirements are met by the visual system, we sought a stimulus that had the following two characteristics (corresponding to the two requirements, respectively): (1) the entities upon which the induced motion effect operates—that is, the target and background motions—should only be available at a relatively late stage in the motion processing pathway. If induced motion occurs as usual in this case then it is very likely to be mediated at a late stage in the motion processing pathway—*after* the target and background motions have been calculated. A scene that is composed of multiple small dispersed motion signals where the signals need to be separated into distinct target and background pools and then each pool integrated to calculate the individual motions of the target and background has the potential to meet this characteristic. Further, if it can be shown that the target and background can be integrated by *functionally separate* mechanisms without changing the induced motion effect then we have even stronger evidence that the induced motion mechanism *follows* the separate integration mechanisms. Further still, the stimulus should allow for the adjustment of low-level aspects of the local elements, such as orientation and spatial frequency, to see if adjusting these low-level properties affects induced motion. A lack of sensitivity to these adjustments would support a higher level mechanism. (2) The shape and position of the background relative to the target should be adjustable without the motions of each causing them to collide or significantly spatially vary relative to one another or vary relative to the fixation point during a trial. This is to allow the testing of what spatial arrangements of the unattended part of the stimulus constitute backgrounds for induced motion.

To this end, a novel induced motion stimulus was designed. It consisted of two sets of dispersed patterned elements; one set for the target and one for the background. Each element remained in place during each trial but the pattern it contained drifted in a way that was consistent with the motions of the other patterns belonging to the same “object”—target or background—as if the stationary elements were windows through which small parts of a larger moving object could be seen; one set of windows for the target and another for the background. Using stationary local elements meant that the background and target shapes, positions and motions could be independently varied without them ever colliding, running off the screen or coming nearer to or further from fixation during a trial.

**Figure 2.**
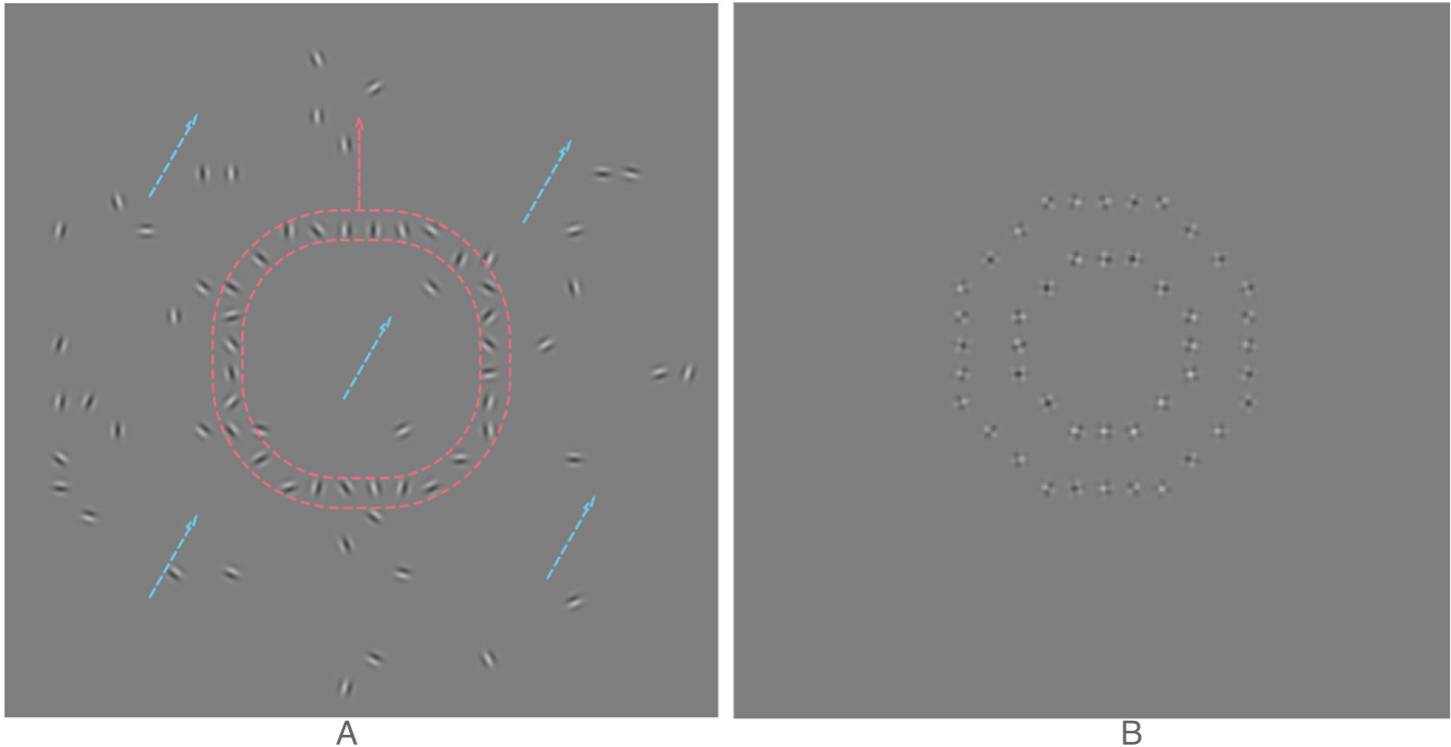
Example stimuli used in our experiments. (A) A representation of the “1D” stimulus used in our first experiment; target ring against a field background. Shown is the central portion of the stimulus display plus dashed lines to indicate the shape of and direction of motion for the target (red) and the background (blue). All Gabor envelopes were stationary but each sinusoidal carrier drifted at a speed consistent with the overall motion of the target or background to which it belongs. Although the actual target object velocity is upwards it appears to move up and to the left. Please see video 2A in online Supplemental Material. (B) An example “2D” stimulus used in the fourth experiment; ring target with ring “background” where the background is **inside** the target. Each small patch stayed in place but the plaid patterns within them drifted so as to evoke a separate sense of motion in the target and background. In the video associated with the image, the outer target ring velocity is upwards, but it appears to move up and to the left as the inner background ring has a rightward component to its velocity. Please see video 2B in online Supplemental Material.

So that we could specifically control the spatial frequency and orientation content of the target and background and so that no single local element could give away the direction of the target or background to which it belonged, most conditions used randomly oriented local Gabor patches with drifting carriers; sinusoidal gratings within circular Gaussian weighting functions (windows) where the phase of the grating drifted with time. Each element’s drift speed was consistent with the rigid motion of the target or background to which it belonged. See Figure 2A and Video 2A in online Supplemental Material for an example stimulus.

Each element in this stimulus provides only a local “1D” picture of the global 2D motion of the object to which it belongs as motion information in the direction parallel to the stripes is undetectable leaving a single explicit motion component—the one orthogonal to the grating stripes. One needs to integrate over at least two differently oriented 1D elements to get a picture of the 2D global motion of the object to which the elements belong. Computationally, the global motion solution lies at the “intersection of [the] constraints” (IOC) introduced by the motion of each local 1D drifting Gabor element (Adelson & Movshon, 1982). Importantly, it has been shown previously that the integration method employed by the visual system when combining local 1D motion signals corresponds to the IOC solution whereas an *averaging* integration method is used if the local 1D signals are replaced with local 2D signals (Amano et al., 2009a; Bowns & Alais, 2006). These constitute two functionally separate integration methods which, if used according to the logic outlined above, can be used to provide strong evidence for a relatively late-stage induced motion mechanism.

To achieve local 2D motion signals using stationary patches a textured pattern needs to drift within each circular window. We used plaid patches, which consist of two overlayed orthogonal gratings within circular Gaussian windows, to produce local 2D motion signals (see Figure 2B and Video 2B in online Supplemental Material).

We define the “target” in our stimulus as the set of local elements to which the observer was instructed to attend in an effort to judge its direction. In all cases this was a set of elements lying on a ring centered on the (instructed) fixation area where the patterns within the local elements moved in a way consistent with a certain global speed and direction which we define as the “motion” of the target. The spatial arrangement of the unattended “background” local elements varied between conditions from field (scattered over the stimulus area including inside the target) to ring-shaped (cf. target) and the background’s “motion” was defined in the same way as it was for the target. We take the directions of the targets and backgrounds defined in this way and compare them with directions of targets and backgrounds in traditional induced motion experiments.

It is important to note that our novel 1D induced motion stimulus requires the visual system to segregate target and background local elements while at the same time calculating the global motions of the target and background. If an induced motion effect, similar to what has been seen in previous studies, can be elicited with this stimulus, even before any other tests are conducted, it would suggest a higher level for the induced motion effect. This is because the target and background motions upon which the induced motion effect rest can only be calculated after local elements have been assigned correctly to the target and background and each set of elements has been integrated to obtain the global target and background motions. Integrated global motion solutions, for both 1D and 2D elements, have been shown to be represented in area MT (Britten et al., 1993; Movshon et al., 1985; Newsome et al., 1989; Smith et al., 2005)—already a mid-level stage area in the motion processing pathway—so the occurrence of induced motion with our stimulus—especially the 1D stimulus where the global 2D motion can *only* be obtained by integration—would suggest that induced motion be mediated at this level at the earliest.

Our first experiment, then, was to test for an induced motion effect using our 1D stimulus. To be sure that any repulsion between the target and background was a result of induced motion and not some other related phenomenon we mapped out the repulsion effect as a function of background direction. Doing so allowed us to discern between two plausible causes for the repulsion, (1) induced motion and (2) the so-called “motion direction illusion” (e.g., Wiese & Wenderoth, 2007) or “direction repulsion effect” (e.g., Benton & Curran, 2003) wherein two interspersed populations of dots (or similar) move in two different directions and the perceived directions of each perceived transparent sheet are repulsed away from one another. Each of these possible causes predicts a different relationship between background direction and the repulsion magnitude. Specifically, the induced motion effect peaks when the background direction is orthogonal to the perceived target direction (Farrell-Whelan et al., 2012), whereas the peak tends to be at significantly smaller separation angles for the direction repulsion effect (see Experiment 1 Discussion section below for an explanation). Accordingly, we compared our results to those from both an induced motion experiment using a more traditional stimulus than ours (Farrell-Whelan et al., 2012) and a classic direction repulsion experiment (Marshak & Sekuler, 1979).

Although the simplest explanation for an induced motion effect in Experiment 1 would be that the effect occurs *after* the integration step, it is possible that there is a much more complicated mechanism that involves interactions *before* or *during* the integration step. The experiments described below were designed to test for this possibility. By eliminating low-level explanations we aimed to show that the first requirement for the flow-parsing hypothesis is satisfied, that is, that induced motion is mediated in a higher level area of the motion pathway.

In Experiments 2 and 3, we tested whether the repulsion effect is changed by significant low-level differences between the target and background. By “low-level” we mean differences that would influence the repulsion effect if it were mediated by lower-level areas in the visual motion system such as area V1 or area MT. Specifically, we tested whether the target/background interaction is changed by a 1-octave difference in spatial frequency content between the target and background (Experiment 2) and whether the interaction is changed when target and background local directions are separated in direction space by 45° (Experiment 3).

We expected that, if the influence of the background on the target were mediated by a low-level mechanism, the strength of interaction between the target and background would be altered by these low-level differences between them. For example, if the effect were mediated by V1 neurons there should be a decrease in the interaction between nearby target and background elements with spatial frequency difference as individual V1 neurons are tuned to this feature and interactions between V1 neurons tend to fall off with separation in preferred spatial-frequency (e.g., Aghajari et al., 2020; Blakemore & Campbell, 1969; Polat & Sagi, 1993) just as they do with separation in orientation preference (e.g., Apthorp et al., 2017; Blakemore & Campbell, 1969; Cannon & Fullenkamp, 1991; Petrov & McKee, 2009). In our tests, the target was composed of elements lying in one spatial frequency (Experiment 2) or orientation (Experiment 3) band and the background was composed of elements in another significantly separated band.

The final psychophysical test for the possibility of a low-level induced motion mechanism involved different combinations of 1D and 2D local elements. If the repulsion effect is implemented after the local motion signals in a scene have been segregated into target and background pools and each pool of signals has been integrated to form an estimate of target and background motion then one would expect the characteristics of the repulsion to be the same whether the scene consisted of 2D motion signals or 1D motion signals. They are each processed by different systems in the visual pathway (Amano et al., 2009a; Bowns & Alais, 2006) but if it is the final result of each system that is fed into the repulsion mechanism then the results should be the same. But note that, even if we confirm their similarity, there still remains the possibility that the 1D motion processing system and the 2D motion processing system each possess similar target/background repulsion mechanisms and that the repulsion is implemented before those signals are fully processed, that is, there is cross-talk between target and background motion signals within the 1D (and similarly 2D) motion processing system that causes the repulsion. So, in Experiment 4, we compared the results of same (1D/1D or 2D/2D) target and background type with different (1D/2D or 2D/1D) target and background type. If the results are the same in both cases we have strong evidence against the effect being mediated by cross-talk *within* integration systems.

The four experiments outlined above address the requirement that induced motion be mediated in a relatively late stage in the motion processing pathway. We addressed the second requirement of the optic-flow explanation—that the unattended part of the scene be treated as a stationary background against which an observer is moving—by running three versions of Experiment 1—each using a different background configuration—and comparing the results. We begin by using a stimulus that is most conducive to an object-moving-against-a-stationary-background interpretation, that is, a ring-like target centered on fixation moving against a dispersed background motion field. We compare the resulting induced motion effect with that from a stimulus where the unattended motion belongs to an object the same shape as the target but surrounding the target. We go further to test the induced motion magnitude when the unattended ring is nested *inside* the target ring. If our results are the same in the three cases then what is considered background in the induced motion effect may simply be a matter of attention; having nothing to do with the shape or spatial arrangement of the unattended motion elements relative to the target; if the motion elements are unattended they are considered background. Whether such a notion of background is conducive with the flow-parsing hypothesis is discussed in the General Discussion.

In the General Discussion, we also apply a simple model based on the flow-parsing concept to both our data and the data of Farrell-Whelan. A close fit of the model would be expected if the visual system does, indeed, implement a flow-parsing mechanism.

In summary of the approach to follow, if the flow-parsing interpretation of the classic Induced Motion Illusion is to hold we need psychophysical evidence that the illusion is mediated at a level in the visual motion pathway capable of optic-flow analysis. We also need to be able to interpret induced motion scene elements within a flow-parsing framework including interpreting the unattended parts of induced motion scenes as stationary backgrounds against which an observer is moving. We explore the background question and provide evidence for a high level—possibly optic-flow stage—mechanism in the four experiments below.

## Experiment 1. Does Our 1D Stimulus (With Various Background Configurations) Produce Induced Motion?

The aim of Experiment 1 was to test for induced motion using our novel 1D stimulus. Specifically, we assessed perceived target repulsion as a function of background direction in order to compare our results with those from a previous induced motion experiment and, for contrast, a direction repulsion effect experiment. Similarity to the previous induced motion experiment would indicate that induced motion occurs at least at the motion processing stage that calculates global motion from local 1D motion signals, as the global target and background motion signals are not available prior to that stage.

The experiment consisted of three conditions: background field, background ring outside, and background ring inside the target ring. Our aim in using different background types was to investigate the strength of the induced motion effect as a function of the extent and shape of the background, specifically, we tested whether object-like backgrounds could be just as effective at inducing motion in the target as field-like backgrounds as suggested by previous literature. If so, could the background be placed inside the target and still be just as effective? No change in the size of the effect would mean the flow-parsing hypothesis needs a very broad concept of what constitutes an optic-flow background if it is to hold; something defined more by attention than physical extent or position.

### Method

For all experiments, the stimuli were presented on a SONY Trinitron G420 monitor (1024 × 768 pixels at 100 Hz) that was placed 60 cm from a chin rest. Images were created in MATLAB R2013b on a PC running Windows 8. The stimuli were stored and presented using a Cambridge Research Systems (CRS) ViSaGe visual stimuli generator. Two buttons located on the top row of a CB6 Response Box (CRS) were used to register participant responses.

All participants gave written informed consent prior to beginning the experiments. These experiments had ethics approval (RA/4/1/4503) from the Human Ethics Committee at the University of Western Australia.

In all three conditions the target consisted of 28 Gabors evenly distributed around a 4° radius ring centered on the display. Observers were instructed to fixate as close to the center of the ring as they could (a fixation point was not included as it would provide an unwanted reference for judging target motion). In the background field condition, the background consisted of 40 Gabor elements randomly scattered over a 20° × 20° region centered on the display (elements were free to also appear *inside* the target ring), in condition 2 the 40 Gabor elements were evenly distributed around a 5.6° ring surrounding the target, and in condition 3 there were 16 Gabor elements evenly distributed on a 2.4° ring inside the target. All Gabor elements were distributed and oriented randomly at the beginning of each trial. The Gaussian envelopes had a standard deviation of 8′, and the carrier had a spatial frequency of 3 cycles per degree (c/°). All Gabor elements in the target ring had a Michelson contrast of 0.40 ([*L*max–*L*min]/[*L*max + *L*min]) or, in the case of the inner and outer background rings, had a contrast that perceptually matched the target contrast. Since piloting showed that this adjustment had little apparent effect the elements in the field also had 0.4 contrast. Each Gabor element’s phase drifted at a rate that depended on its orientation and the speed and direction of the target or background object to which it belonged. For example, if the Gabor element was part of the background, its rate of drift was the dot product of a unit vector representing the direction of drift (orthogonal to the Gabor’s stripes) and a vector representing the speed and direction of the background. This is equivalent to making all background Gabor elements drift consistently with the IOC solution for the desired background motion. The target was separately constructed in the same manner. This produced the percept of two rigidly moving objects, one being the target and the other the background. Both “moved” in this way at a speed of 6^o^/s.

There were two versions of the background ring outside condition; one for making a comparison with the background field condition and the other for making a comparison with the background ring inside condition. They are denoted, respectively, “background ring” and “background ring outside.”

For each background field versus background ring session the background moved in one of five randomly chosen directions clockwise from vertical: 0°, 15°, 30°, 60°, or 90°. A moving target was simultaneously presented with the background on each trial and participants indicated whether the target appeared to move clockwise or anticlockwise of vertical. The target directions were chosen and responses were analyzed in real time using the Psi Psychophysical Method (Prins & Kingdom, 2018) in order to find the point of subjective vertical (upwards drift) for the target for each background direction. Seventy five trials were run during each background direction session as pilot studies showed that threshold estimates tended to be stable at this point and each participant did each background direction session twice. Background field and background ring sessions were randomly interleaved. Each stimulus was presented for 500 ms and a central fixation point appeared between stimulus presentations.

For the background field and background ring conditions, participants consisted of five experienced observers recruited from the Vision Lab at the University of Western Australia. All participants except KH were naïve to the hypothesis of the experiment. All participants had normal or corrected-to-normal visual acuity. Observer ED has a divergent squint and completed the experiments using an opaque eye patch over the non-dominant eye.

For background ring outside and background ring inside conditions, the background direction was always 30° and the subjective vertical direction for the target was found using the same method as for background field and background ring conditions. As we wanted to compare background outside with background inside the target ring, we needed to control for perceived contrast as a function of eccentricity as it has been shown previously that contrast detection thresholds vary with eccentricity (Koenderink et al., 1978a, 1978b), perceived speed can vary with perceived contrast (Thompson, 1982), and signal segmentation can occur as a result of differences in apparent contrast (Croner & Albright, 1997).

Each participant, therefore, first completed a series of contrast matching sessions—one for each of the eight conditions described below—and their results were used to normalize the apparent contrasts for each participant individually in following sessions. During contrast matching sessions, participants indicated whether the inner or outer ring—one being the same radius as the target ring—was of higher contrast and the Psi Psychophysical Method was used to find the contrast for the non-target ring which equated the appearance of the two rings. Stimulus presentation time was the same as for the main experiment.

Background ring outside and background ring inside conditions were run in conjunction with Experiment 2 outlined below using the same set of participants. Accordingly, there were eight conditions. Each was run twice for each participant. The conditions were randomly interleaved. The eight conditions were all possible combinations of the following pairs: background inside/outside of target (Experiment 1, part 2 conditions), target high spatial frequency/low spatial frequency, and background high spatial frequency/low spatial frequency (Experiment 2 conditions).

Eight participants took part in these experiments. ED, TM, and DP were experienced observers. All participants, except JH, were naïve to the hypotheses of the experiments. Participants all had normal or corrected to normal visual acuity. Observer ED has a divergent squint and completed the experiment using an opaque eye patch and monocular vision.

### Results

**Figure 3.**
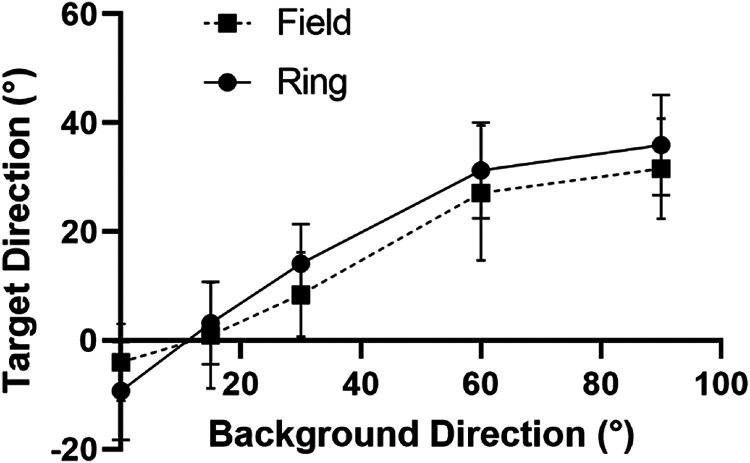
Experiment 1 part 1; background field versus background ring. Shown is target direction that is perceived as vertical for a range of background directions for both the background ring and background field conditions. Both are measured in degrees clockwise of vertical. This convention is used in all graphs to follow. The averaged performance of the group of observers is plotted. Error bars represent 1 *SD*.

In the first part of this experiment, the stimulus consisted of a ring target moving in an IOC-defined direction against either a background field of randomly positioned Gabors or a ring of Gabors moving with a different IOC direction—the motion being produced by physically stationary “drifting” 1D motion elements as just described in the Methods section. The deviation of the target direction clockwise from vertical while being perceived as vertical is plotted against the background direction (also in degrees clockwise from vertical) in Figure 3.

Note that the axes represent the direction of the background (horizontal axis) and the direction of the target (vertical axis) motion relative to vertical in a clockwise direction, that is, 0 means vertically upwards and 90 means horizontally rightwards motion. It is plotted in this way so that the vertical axis is indicative of the size of “repulsion” effect, that is, the deviation of the target from vertical while still appearing vertical. This convention for the vertical axis is used for all plots below.

There was a similar pattern of results across observers but there are large differences in effect size between observers. This is reflected in the large standard deviations in the combined results shown in Figure 3 (note, 95% CI not shown as the significance of the means resulting from the spread between observers is not the focus). Individual results are shown in the Supplementary section for the interested reader. The mean target direction that appears vertical when the background direction is also vertical is non-zero for both data sets but the difference from zero is non-significant for both the field (*t*[4] = 1.26, *p* = .28, two-tailed *t*-test) and ring (*t*[4] = 2.31, *p* = .08, two-tailed *t*-test). This is discussed further in the Supplemental Section for the interested reader.

The shape of the two curves in Figure 3 is consistent with the repulsion being an induced motion phenomenon. Specifically, the general shape matches that seen in previous induced motion studies (Farrell-Whelan et al., 2012) and doesn’t match those in previous motion direction illusion studies (Marshak & Sekuler, 1979). This important finding and its implications are discussed in more detail in the Experiment 1 Discussion section below.

When the background field of randomly placed Gabors was replaced with the same number of Gabors lying on a ring outside of the target ring, the results were similar. There is no statistically significant difference between having a ring and a field as background (two-way repeated-measures ANOVA, *F* [1, 20] = 2.198, *p* = .154). Giving a shape to the background, that is, making it object-like without changing the number of local Gabors, doesn’t significantly affect its repulsive influence on the target.

**Figure 4.**
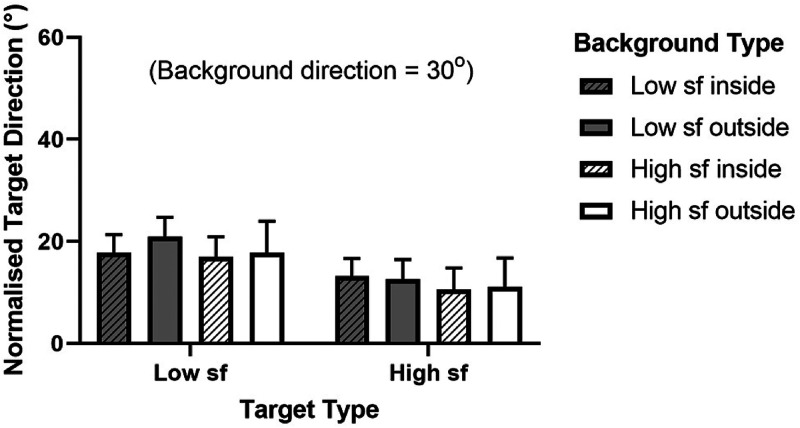
Experiment 1 part 2 and experiment 2; background ring inside versus outside of the target ring with high and low spatial frequency (“sf”) local elements. The target direction normalization process is described in the text. Error bars represent 95% confidence intervals.

We also tested whether the position of the background relative to the target, inside or outside, made a difference to the induced motion effect. The group-averaged results of this comparison are shown in Figure 4 along with the results of Experiment 2. Having the background ring outside versus inside the target ring is compared for both low spatial frequency and high spatial frequency target and background Gabors. To examine the effect of having the background inside versus outside of the target simply compare directly-neighboring “inside” and “outside” Background Type conditions in the graph; the two striped bars in each set of four are Inside conditions and the other two are Outside. The background direction was 30° clockwise from vertical for all conditions as explained in the Methods section.

Despite the similar pattern of results across participants there were large differences in actual target repulsion magnitudes. For this reason, the data was normalized to remove differences between individuals using two baseline conditions, that is, all “background outside” conditions were normalized using the target low frequency/background low frequency outside condition (as it matched our first experiment) and all “background inside” conditions were normalized using the target low frequency/background low frequency inside condition. We used an additive normalization process (new result = mean baseline + [old – individual baseline]). The background outside and background inside conditions were normalized separately because the direction of the biases for each participant varied according to the position of the background. Note that in normalizing the data the group means did not change, only the variance in the group data was affected, that is to say, a graph of the raw data would be identical to the normalized one but error bars would be larger and the pattern of differences between conditions would be the same.

The position of the background relative to the target—inside or outside—had no significant effect on the repulsion interaction between target and background (three-way repeated measures ANOVA, *F* [1, 56] = 0.1325, *p* = .717). The RMANOVA was conducted on the raw data using the factors target frequency, background frequency, and background position.

### Discussion

The primary goal of Experiment 1 was to see if our novel dispersed 1D local motion stimulus produced an induced motion effect. If the characteristics of the target repulsion seen here compare well to those from previous induced motion experiments it would indicate that induced motion is mediated in a brain area that follows the local element segregation and integration steps as the target and background motions upon which the effect acts are not available until after those steps. Accordingly, we compared our results to those from Farrell-Whelan et al. (2012), who performed a standard induced motion experiment, and, to make sure our results were not consistent with the direction repulsion effect, we plotted our results alongside those of Marshak and Sekuler (1979), whose study is considered a classic direction repulsion experiment.

**Figure 5.**
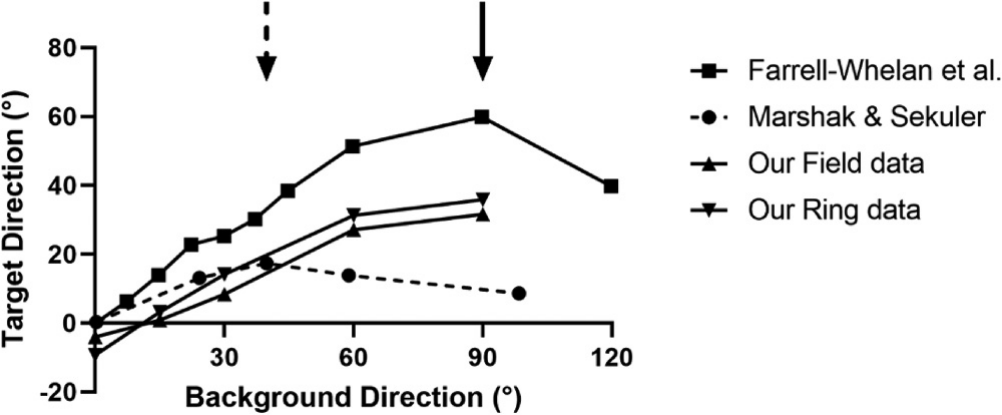
Comparison of our results with those of Farrell-Whelan et al. (2012) and Marshak and Sekuler (1979)—the first being an induced motion experiment and the second a direction repulsion experiment. Note that both the Target Direction and Background Direction are measured relative to the perceived target direction. The dashed arrow points to the peak of the Marshak curve and the full arrow points to the peak of the Farrell-Whelan curve. Pilot studies indicated that our repulsion effects peaked in the same place as Farrell-Whelan. Peaking at 90° is a feature of induced motion (see text). Data taken from Experiment 1 above, (Farrell-Whelan et al., 2012) and (Marshak & Sekuler, 1979).

Both teams looked at the repulsion strength as a function of the directional separation between the target and background. One important difference between the two effects is clear when the repulsion strength is plotted against the background direction *relative to the perceived target direction*. Farrell-Whelan et al. (2012) measured this directly but for Marshak and Sekuler (1979) the background direction relative to perceived target was, here, calculated by adding the perceived deviation of the target from its actual direction to the difference between the target and background direction. Like Farrell-Whelan et al. (2012) we measured this directly by setting the perceived target direction to vertical and measuring the effect of the background direction relative to vertical. Figure 5 shows the result of the comparison.

For induced motion, the effect size increases with the speed of the background (Gogel, 1979; Post & Heckmann, 1986), albeit at a diminishing rate (Wallach & Becklen, 1983).^1^ The maximum induced motion effect corresponds to the point at which the target direction is most deviated from the perceived target direction, that is, if an axis is drawn that is orthogonal to perceived target direction, the actual target component on this axis is at its maximum. This will occur when the background component on that axis is also maximal. This occurs at 90° on the *x*-axis in Figure 5 because, for a fixed speed background, its speed component orthogonal to the perceived target direction is maximum when the background is orthogonal to the perceived target direction. The relationship of the effect size to speed of background is not so straight-forward for the direction repulsion effect (Curran & Benton, 2003) so the peak need not lie at 90° (Mather & Moulden, 1980). Our pilot studies clearly showed a drop off in effect size after 90° but, to minimize an already-large observer load we chose to measure only up to the peak. Our results are qualitatively similar to those of Farrell-Whelan albeit diminished in strength. This demonstrates that the effect we are seeing in our results is consistent with the induced motion effect. Consequently, this experiment provides evidence that induced motion is mediated in a relatively late stage of the motion processing system—following the 1D motion integration system that works out the motions of the target and background.

Our results also demonstrate that the induced motion effect is insensitive to significant changes in the shape and position of the background relative to the target. Even having the background the same shape as the target and positioned inside it didn’t change the effect size. This confirms the results of some other researchers that a background need not surround or be larger than the target (e.g., Carr & Hardy, 1920; Day et al., 1979; Duncker, 1929). It also suggests that what constitutes background is a matter of attention; in the background-inside and background-outside conditions both the target and background were rings and the only constant feature of the background was that it was the ring not being attended to. This calls for a more abstract definition of what constitutes background in induced motion experiments (contrast with, e.g., Palmer, 1999) and requires just as broad a definition for what constitutes an optic flow background if the flow-parsing explanation of induced motion is to hold. This is discussed further in the General Discussion section.

The fact that the background-inside results matched those of the background-outside condition also invalidates a simple attentional spotlight mechanism wherein a circular “spotlight” is shone on a retinal area that covers the target and the target’s motion is calculated by integrating signals within that area. If that were the case, for the background-inside condition, the background motion elements would be integrated into the target and a target motion somewhere between the true target and background motions would be used as input into the induced motion system leading to a different set of perceived target directions than those for when the background was outside of the spotlight. This would likely lead to lower repulsion effects. There was no such difference indicating that the visual system was capable of correctly segregating target and background elements independent of where they were placed in the scene.

These results also speak to our aim of uncovering the processing stage at which induced motion occurs. We have demonstrated that what constitutes background in induced motion experiments is far from simple; too complex to be easily compatible with a simple low-level mechanism. Attention-driven feedback from higher areas to a theoretical lower-level induced motion mechanism where the feedback tells the system what is background and what is target is possible, but a simpler solution is to have the induced motion mechanism follow the integration system that determines what is target and what is background. Also, V1-level interactions are likely to be local—occurring between close-neighboring Gabor elements—and the average number of close target-background neighbors is much greater for the background ring conditions than for the background field condition where the background elements are dispersed. We saw no change in the induced motion effect between these conditions.

## Experiment 2. Does Spatial Frequency of Background Relative to Target Matter?

There are three possible ways the target and background elements were interacting to produce the effects seen so far. The first is that the individual elements were interacting before they were segmented and integrated into target and background global percepts, the second is that they were interacting during the integration phase, and the third is that they were interacting afterwards, once the global target and background solutions were reached. The first is unlikely as individual elements do not contain information on their own about the target and background motions and we are seeing interactions that are consistent with induced motion based on the global target and background IOC solutions. But if the interaction were somehow occurring at this low processing level (probably V1), then we would expect to see a decrease in interactions between nearby target and background elements the more they differed in spatial frequency (e.g., Blakemore & Campbell, 1969; Cannon & Fullenkamp, 1991; Kim & Wilson, 1997). In Experiment 2 we used a 1 octave difference which, by low-level explanations, should result in a minimal interaction between the target and background elements (Blakemore & Campbell, 1969; Cannon & Fullenkamp, 1991). If the second type of interaction (in the integration phase) were occurring, then the magnitude of interaction is also likely to be sensitive to spatial frequency based on work conducted previously in our lab. Amano et al. (2009b) tested the spatial frequency tuning of the integration systems for Global Gabor stimuli like the ones used in our experiments by looking at how noise elements of one spatial frequency influence the threshold of detection for global motion carried by elements at another spatial frequency. Thresholds dropped approximately three-fold on average for one octave differences between signal and noise elements for 0.7 and 1.4 cycle/deg signal elements and the trend appeared to be the same for 2.8 cycle/deg signal elements (although testing didn’t occur for 5.6 cycle/deg noise elements). Extrapolating these results, we’d expect a low magnitude of interaction between low spatial frequency (3 c/°) and high spatial frequency (6 c/°) elements during the integration stage; much lower than for elements of the same spatial frequency. Our aim was to test for the decrease predicted by both low-level explanations.

### Method

In this and all remaining conditions the background moved at a direction of 30°. This direction was chosen as our Experiment 1 results showed it was far enough from vertical to produce a reliable significant deviation in perceived target direction from vertical. As outlined above, the Experiment 2 conditions were run in conjunction with Experiment 1 Part 2 to facilitate comparison making a total of eight conditions. In order to assess the influence of spatial frequency differences between the target and background, the target elements were either high or low spatial frequency and the background either high or low. “Low frequency” Gabor elements had a spatial frequency of 3 c/° (the standard spatial frequency used in our other experiments) and “high frequency” Gabor elements had a frequency of 6 c/°; a one octave difference. The target was a ring with radius 4° and, having established that a background ring was just as effective as a field at driving induced motion, the background was also a ring, but with a radius of 5.6° or 2.4° as described in Experiment 1. There were eight participants as described above. The session methods and procedures were the same as those described for Experiment 1.

### Results

Our results for low spatial frequency and high spatial frequency target and background combinations are depicted alongside the results of Experiments 1 part 2 in Figure 4 since these conditions were run in parallel. For a given target type, compare high (white bars) and low (grey bars) frequency background results. In our experiments, the spatial frequency of the background for a given target frequency had no significant effect on the repulsion interaction between target and background (three-way repeated measures ANOVA, *F* [1, 56] = 0.6185, *p* = .435 for main effect of background frequency and *F* [1, 56] = 0.0006, *p* = .981 for target/background frequency interaction). As above, the RMANOVA was conducted on the raw data using the factors target frequency, background frequency, and background position.

## Experiment 3. Does Orientation Content of Background Relative to Target Matter?

As our targets and backgrounds, up until now, have been composed of randomly oriented elements, it is possible that interactions are strong between similarly oriented neighboring components and weak or non-existent between differently-oriented neighbors and that we are seeing an average of these interactions. This would be conducive with a low-level explanation of the effect as low-level interactions tend to be localized in retinal space (e.g., Badcock & Westheimer, 1985; Kapadia et al., 2000; Polat & Sagi, 1993) and tend to decrease in strength as orientations differ between components (e.g., Apthorp et al., 2017; Blakemore & Campbell, 1969; Cannon & Fullenkamp, 1991; Petrov & McKee, 2009). To test for this, we compared the induced motion effect when the orientation content of the target and background were separated by 45° with the effect obtained when the orientation content of the target spanned the same space as the background (a full 360°). Specifically, we were looking for a decrease in the target/background interaction with an increase in the smallest differences between local orientations. For most low-level orientation interactions strengths are weak to non-existent with a 45° difference between element orientations (e.g., Apthorp et al., 2017; Cannon & Fullenkamp, 1991) so one would expect to see significantly lower magnitudes of induced motion if the effect were mediated in low-level visual areas.

If the interaction were occurring during or after the integration stage we would expect no change with orientation difference as it was shown by Amano et al. (2009b) that interactions during integration are not tuned to the orientations of the local elements.

Note, also, that differences of up to 45° between local elements normally produce weak tilt illusion effects (Dickinson et al., 2010, 2012; Takao et al., 2020). In this condition that influence was constant unlike in the 360° condition.

### Method

Participants each took part in two sessions corresponding to the two conditions twice. The session methods and procedures were the same as those described above. There was no need to control for perceived contrast of the background as we were not comparing a background ring outside to a ring inside of the target ring; the 40 background elements were randomly scattered across a field just as in the first Experiment 1 condition.

In order to produce a condition where the orientation content of the target and background were separated by 45°, each background element’s orientation was randomly assigned to one of the cardinal axes, and the target element’s to one of the intercardinals.

Five participants took part in this experiment. All were experienced observers and all but DB and MF were naïve to the hypotheses of the experiments. Participants all had normal or corrected to normal visual acuity. Observer ED has a divergent squint and completed the experiment using an opaque eye patch and monocular vision.

### Results

There was no statistically significant difference between the induced motion effects for the two conditions for the group (paired, two-tailed *t*-test, *p* = .23). The mean difference was 2.46° with a 95% CI of 4.79°. Nor was there a statistically significant difference for any individual observer (*p* > .05).

## Experiment 4. Does Element Type (1D or 2D) Matter?

If the induced motion effect is unaffected by particular combinations of 1D and 2D targets and backgrounds, then the effect is very likely to be mediated after the separate 1D and 2D integration systems as explained in the Introduction section.

As in Experiments 1 and 2, we positioned the background ring inside or outside of the target at the same time as testing for the effect of using 1D and 2D targets and backgrounds.

### Method

The target and background were contrast matched using the method described above to control for potential contrast effects of the position changes. Eight conditions were used which represented all possible combinations of the pairs: background inside/outside of target, target 1D/2D, and background 1D/2D.

A 1D target or background was made up of Gabor elements just as in previous experiments. A 2D target or background was made up of plaid patterns instead. Each plaid pattern consisted of the sum of two orthogonal 3 c/° gratings set within a Gaussian envelope and drifted at the speed and direction of the target or background to which it belonged. Its orientation was random just as with the 1D Gabors. For both the Gabors and the plaid patterns the target elements had a Michelson contrast of 0.4 (i.e., 0.2 for each of the plaid components). The target ring radius remained 4° and the two background ring radii were 2.4° and 5.6° as in Experiments 1 and 2.

Four participants took part in this experiment. All were experienced observers and all but DB were naïve to the hypotheses of the experiments. Participants all had normal or corrected to normal visual acuity. Observer ED has a divergent squint and completed the experiment using an opaque eye patch and monocular vision.

### Results

**Figure 6.**
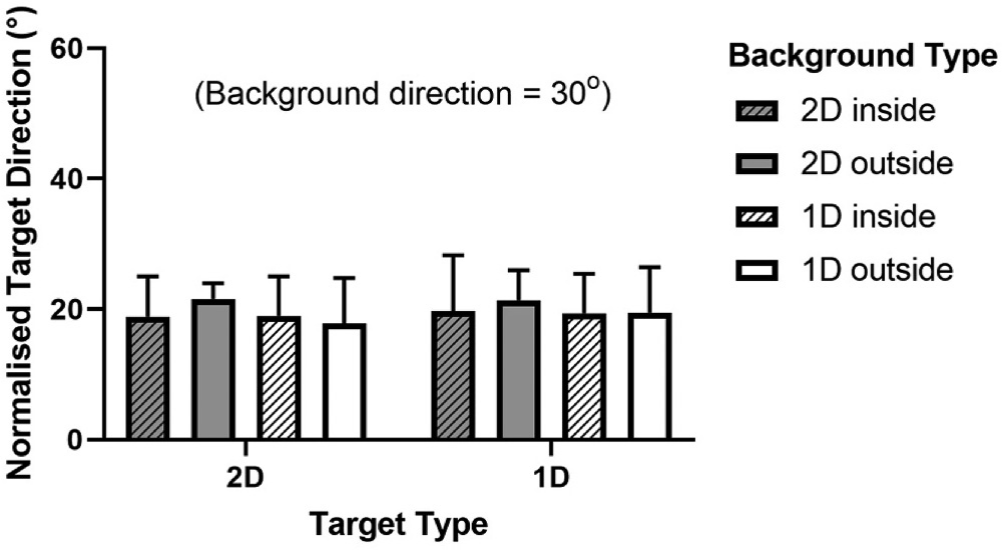
Experiment 4; comparing background and target types—1D and 2D—along with background relative position. The target direction normalization process is described in the text. Error bars represent 95% confidence intervals.

*Note*. 1D = one-dimensional; 2D = two-dimensional.

Figure 6 shows the results of assigning plaids (2D) and Gabors (1D) to the two stimulus components in various combinations; 2D target with 2D background, 2D target with 1D background, 1D target with 2D background, and 1D target with 1D background. At the same time, having the background ring inside versus outside of the target is compared to verify Experiment 1 results. Directly comparing Gabor and plaid background types for each target type reveals whether matching 1D with 1D, and 2D with 2D as in standard induced motion experiments produces different results to having mixed target and background types. The data was normalized in the same way and for the same reasons listed for Experiment 1 part 2 using the conditions target 1D/background 1D outside and target 1D/background 1D inside as normalizing conditions. Again, this did not alter the means—only the variation in the data.

The repulsion effect is unaffected by background type (three-way RMANOVA main effect of background type, *F* [1, 24] = 0.1458, *p* = .706) nor is there an interaction between target type and background type (*F* [1, 24] = 0.008104, *p* = .929) using the raw data.

### Discussion

Our results indicate that the induced motion effect is the same when the target and background elements are of inconsistent type (one 1D and the other 2D) as when they are consistent (both 1D or both 2D). This means that the induced motion effect is highly likely to be mediated in a brain area that follows the separate 1D and 2D integration pathways in the visual processing stream. As the induced motion effect relies on an interaction between target and background motions, and knowing the target and background motions require integration of the local motion elements, if the interaction is the same no matter what combination of integration channels are used, then it must be the final outputs of the integration channels that are fed into the induced motion system, so that system must lie after those integration channels.

## General Discussion

Our goal was to conduct psychophysical tests of the plausibility of a flow-parsing explanation for the induced motion effect. In particular, we conducted tests to determine the minimum visual processing stage at which the repulsive interaction between target and background occurs as the flow-parsing proposal requires a late-stage interaction, that is, the level at which optic flow is processed. Our results clearly support an interaction occurring *after* the stage at which (both 1D and 2D) local motions are integrated to produce global 2D motion solutions. We found no evidence for an interaction of the sort that produces induced motion at earlier stages. Secondly, we tested the effect of various configurations for the unattended motion in the scene as the flow-parsing proposal requires the unattended motion to be treated as motion belonging to a stationary background against which an observer is moving. We found that the induced motion effect is robust to changes in background configuration—even remaining unchanged when the background was the same shape as the target and placed *inside* the target. We discuss, below, whether these findings are conducive with the flow-parsing view.

In regards to the level at which induced motion is mediated, our results provide support for a three stage process for the effect when a scene is composed of scattered local motion signals:

1. Segregation of local motion signals into target and background pools,
2. separate integration of signals within the two pools and
3. a target/background interaction where background motion is subtracted from target motion.

Ignoring previous studies, it is feasible that steps 1 and 2 occur separately—segregation occurring based on attending to the ring-shaped target followed by separate integration of target and background pools—but it is equally feasible that integration of local signals and segregation into two pools occurs simultaneously via a “conversation” between brain areas leading to a viable interpretation of the scene (Braddick, 1993). The question of simultaneity of these two steps is beyond the scope of this paper. What we argue, given the evidence here, is that the step 3 necessarily occurs after the first two and, thus, at a relatively late stage in the motion processing pathway.

Warren and Rushton (2009) clearly demonstrate the inadequacy of low-level explanations for the shift in perceived target direction, at least in the case when the target moves against an optic flow background. In the case where expanding radial optic flow is restricted to one hemifield and the moving target is placed in the opposite hemifield against a blank background, a low-level explanation would predict that the target direction shift would be away from the center of the display opposite to the prevailing direction of the visible expanding field. Instead, they found that the target direction shift was *toward* the center of the display. This is what would be expected if the *global* motion were used as the background for the target. In this case the expansive optic flow, if projected to the area near the target, would be away from the center of the display so the result that was actually seen would be expected; a shift toward the center of the display. This, too, provides evidence for the locus of the repulsion effect being modulated in a higher visual area that deals with optic flow.

Note, though, that a psychophysical study by Harris and German (2008) indicates that the induced motion effect is implemented prior to the placing of visual motions into a 3D world model. In a stereoscopic display of (1) motion in depth and (2) lateral motion, the induced motion effect (perceived motion of stationary target as fraction of background motion) was the same in terms of the perceived retinal motion of the target and retinal motion of the background, but different when cast in terms of the motions in the real world implied by the display. This may indicate that induced motion falls out of a rough, first-pass estimator of target motion based on retinal motion alone (see also Brenner, 1991; Harris et al., 2004). This is in contrast to work by Warren and Rushton (Rushton & Warren, 2005; Warren & Rushton, 2007) showing that the depth of the target relative to moving background elements is taken into account when judging target motion in 3D scenes. The fact that depth information *can* be ignored indicates that the repulsion effect on the target may *first* be calculated at a level in the motion processing pathway that doesn’t represent a full 3D interpretation of the visual scene.

Taken together with our results, this places rough bounds on the location of the induced motion mechanism in the motion processing pathway. Area MST in the monkey (and the equivalent in humans) is a good candidate as it lies after area MT where the result of the 1D and 2D integration processes is thought to be represented (Smith et al., 2005), it is insensitive to the low-level spatial differences between the target and background we used in our experiments (Duffy & Wurtz, 1991; Geesaman & Andersen, 1996), it is capable of representing optic flow as per Warren and Rushton’s proposal (Wurtz, 1998), and does not appear to fully encode 3D motion information (Héjja-Brichard et al., 2020). Further support for an MST-mediated effect can be found in (Sasaki et al., 2017, 2019; Takemura et al., 2012; Wild, 2018). Note that this differs from the motion repulsion mechanism which is likely to be a result of mutual inhibition between direction-tuned neurons in lower level motion-selective visual areas (Blakemore et al., 1970; Chen et al., 2005; Hiris & Blake, 1996; Kim & Wilson, 1996; Marshak & Sekuler, 1979; Rauber & Treue, 1999; Wilson & Kim, 1994). Benton and Curran (2003) provide strong evidence that this lower area is likely to be MT as the strength of the effect is a function of the average speed of multiple local moving elements which is calculated in area MT.

**Figure 7.**
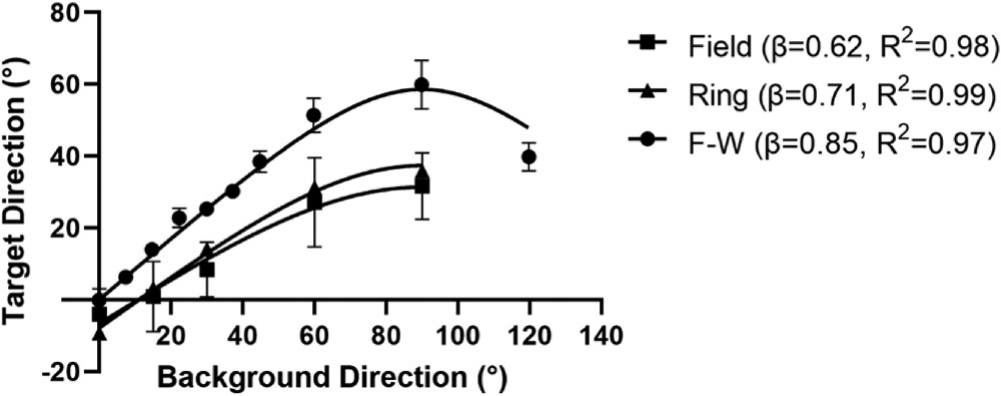
Simple flow-parsing inspired model applied to our data and that of Farrell-Whelan et al. (2012) (F-W). See text for details.

As a final test of the validity of a flow-parsing explanation, we apply a simple model based on the concept. The model is nothing more than a mathematical representation of the flow-parsing process depicted in Figure 1 except that the subtraction represented in the central panel of the figure is only partial. This is required because our results above, and those of previous researchers demonstrate that the background motion subtraction is rarely complete. Accordingly, terms such as “magnitude of induced motion” (Harris & German, 2008; Post & Heckmann, 1986; Schulman, 1981), “extent of induced motion” (Gogel, 1979), “strength of the illusion” (Zivotofsky, 2004), and “measure of induction” (Bassili & Farber, 1977) in the induced motion literature and the “flow parsing gain” (Dupin & Wexler, 2013; Niehorster & Li, 2017) in the target-against-optic-flow literature have been invented to portray the incompleteness of the background subtraction. Our model is represented by the following equation.

p=t−βb

where **p** is a vector representing perceived target motion, **t** is a vector representing actual target motion, **b** is a vector representing background motion, and *β* is the weight given to the −**b** term. The first term can be taken to represent the perceived target motion if there was no self-motion, that is, just the actual target motion across the display, and the second can be thought of as a background-used-as-reference effect (opposite to background motion). *β* controls the weight given to the background reference effect when determining target motion. According to the flow-parsing account, there should be more weight given to this effect if the sense that the background motion is due to self-motion is stronger. In Figure 7, we apply the model to our results as well as those of Farrell-Whelan et al. (2012) shown previously in Figure 5. The fit was produced using GraphPad Prism’s nonlinear regression method employing a least-squares criterion. Included was a variable that allowed for the vertical offsets seen in our data in Figure 5 resulting from biases in the perception of vertical when the background direction was vertical.

*R*^2^ values for the fits to the data were 0.98, 0.99, and 0.97 for our field, our ring, and Farrell-Whelan’s data, respectively. The fact that such a large proportion of the variation in the data can be explained with this simple equation makes a percept based on a mixture of the actual target motion and motion resulting from a flow-parsing construct a viable explanation. The larger *β* value for the Farrell-Whelan data may be a result of a reduced sense of the *actual* motion of the target due to the use of a black tube through which the stimulus was viewed (removing peripheral cues) as Zivotofsky (2004) used a very similar stimulus to Farrell-Whelan but without the tube and got similar *β* values to us. According to the flow-parsing hypothesis, this would mean a higher tendency to assign background motion to self-motion in the absence of peripheral visual cues indicating that the observer was stationary.

What is curious, given the data pointing to induced motion being, at least partially, an optic-flow level phenomenon, is that “backgrounds” defined at this level can be as simple as a single dot (e.g., Carr & Hardy, 1920; Duncker, 1929) or as abstract as an object the same shape as the target placed *inside* the target (Experiment 2 above). It is viable that a system for determining self-motion be highly flexible, for example, if walking in a darkened environment with only a few spots of light they ought to be usable for navigation/postural stability and when the only view of the world is via a window this “background inside a target” should provide a valid cue for self-motion (indeed, when we sit in a train and see a train next to us moving through a window it is not unusual to get the false impression that we are moving). But by what means do moving object-like components of a scene, which have been traditionally thought to activate an “object motion” pathway (Eifuku & Wurtz, 1998; Rosa & Tweedale, 2001; Tanaka et al., 1986), effectively “jump ship” and activate the self-motion/optic-flow pathway (Britten & Van Wezel, 2002; Britten, 2008) instead? As demonstrated here, attention has the ability to drive the switch.

With traditional 2D-induced motion stimuli, there are no motion-in-depth cues such as expansive flow fields to tag the unattended motion as belonging to a background against which an observer is moving. Conceivably, the targets and backgrounds could be treated equally—as moving objects—by the visual system and by attending to one, the other might be shunted to an optic-flow system. For example, both objects could be represented in area MT in the monkey (Britten et al., 1996; Czuba et al., 2014) then, either, the attended representation is held there while the unattended activates area MSTd which, in turn, sends feedback signals to MT which modifies the perceived motion of the attended stimulus (Layton & Fajen, 2016) or both the attended and unattended signals activate MST but the unattended specifically activates MSTd which is specialized for optic-flow/self-motion and the other activates MSTl which appears to be specialized for representing object motion under conditions of self-motion (Eifuku & Wurtz, 1998; Sasaki et al., 2019). Given the flexibility of area VIP to represent object motion (in the presence of optic flow and other self-motion cues) relative to any task-relevant reference frame (Sasaki et al., 2020), it may act as final judge of object motion as induced motion may be considered the (incorrect) perception of target in reference to the moving background.

We have provided substantial psychophysical evidence for a relatively late-stage mechanism for induced motion by showing that the affect is unaffected by significant low-level differences between local target and unattended element features and by showing that when the target and unattended background are composed of local elements that are integrated via different mid-level mechanisms, the effect is the same. This result is conducive with a flow-parsing explanation of induced motion which requires targets and unattended backgrounds to be treated as (1) objects and (2) backgrounds against which a person is moving, respectively, that is, requires the effect to be mediated in an area of the visual motion pathway that deals with optic flow. We confirm earlier reports that what is considered background is a matter of attention; the unattended motion in the scene is taken as background no matter what physical space it occupies relative to the target. Note, though, that the close fit of the data to our simple model implies *partial* flow-parsing as the background motion subtraction is incomplete, presumably depending on the extent to which the background motion is considered to be due to self-motion.

## Supplemental Material

sj-docx-1-ipe-10.1177_20416695221118111 - Supplemental material for The induced motion effect is a high-level visual phenomenon: Psychophysical evidence Click here for additional data file. Supplemental material, sj-docx-1-ipe-10.1177_20416695221118111 for The induced motion effect is a high-level visual phenomenon: Psychophysical evidence by Michael Falconbridge, Kassandra Hewitt, Julia Haille, David R. Badcock in i-Perception

## Notes

1. Note that both Gogel (1979) and Wallach and Becklen (1983) used the orientation of the perceived path of a vertically moving target surrounded by a horizontally moving background as their measure of induced motion. For Gogel, the orientation barely changed with a proportional increase in background and target speed meaning a constant proportion of the (negative of) background motion was being added to the target, that is, induced motion was proportional to background motion. For Wallach’s higher speeds induced motion similarly increased with background motion but less so as speeds increased.
