# Supplementary material for: The induced motion effect is a high-level visual phenomenon: Psychophysical evidence
Source: Iperception. 2022 Sep 7;13(5):20416695221118111. doi: 10.1177/20416695221118111 (PMC9459461; doi:10.1177/20416695221118111)
Supplement: sj-docx-1-ipe-10.1177_20416695221118111 - Supplemental material for The induced motion effect is a high-level visual phenomenon: Psychophysical evidence [file sj-docx-1-ipe-10.1177_20416695221118111.docx]

**Supplementary Material**

**Experiment 1, Individual Data**

Plotted in Figure 8 is Experiment 1 data showing individual results.

As in (Zivotofsky 2004) the pattern of results is similar for each individual but there are significant differences between individuals. In accordance with the flow-parsing model we postulate that this is due to differences in scene interpretation, specifically, a person’s tendency to assign background motion to self-motion. As our observers were stationary this may equate to differences in cue reliance; vestibular (suggesting the observer is stationary) versus visual (suggesting the observer is in motion). The exact cause of the differences is an area of future research.


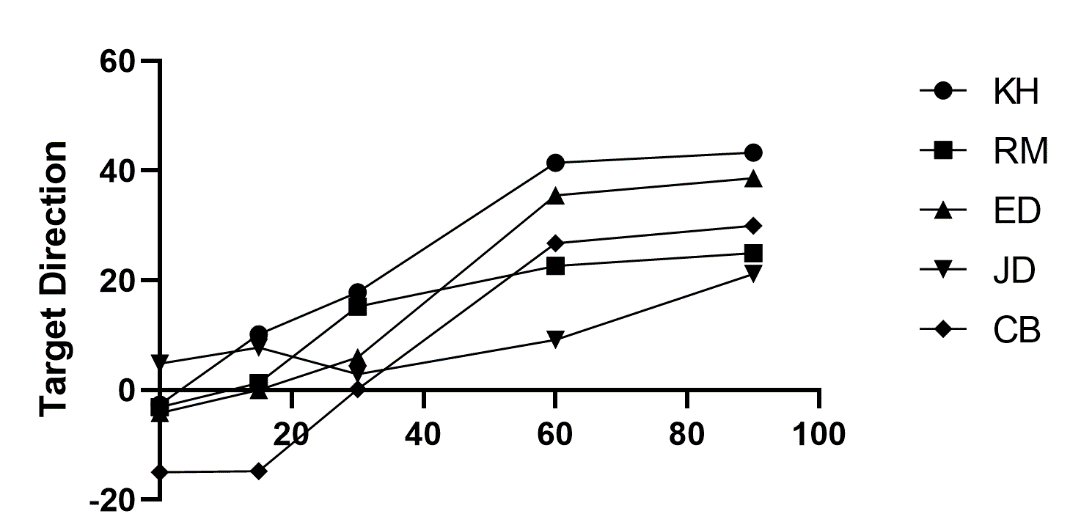

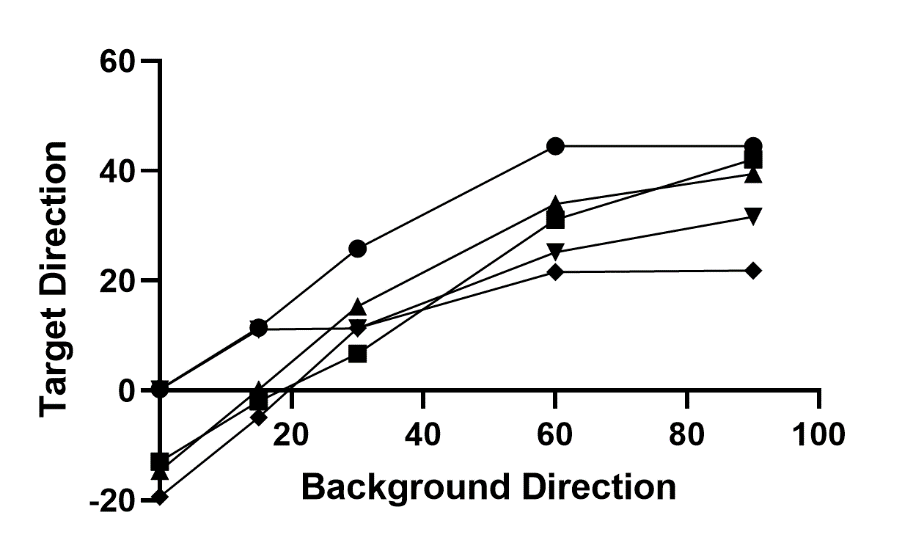


Figure 1 Experiment 1 individual data; target direction that is perceived as vertical for a range of background directions plotted separately for each observer. Each data point represents the average output of two testing sessions. Note that axes represent direction clockwise from vertical in degrees. Top: Background field condition. Bottom: Background ring condition. Directions in degrees.

An interesting trend in the data is a tendency to view targets moving slightly anti-clockwise of vertical as moving vertically when the background is moving vertically (see the y-intercepts in the figures above). As stated in the main document, this tendency is non-significant. As it has no bearing on our results (we were interested in whether the pattern of results depicted in these graphs matches the pattern seen in previous induced motion experiments) we did not pursue this tendency. More observers would be needed to see if the effect is significant and still further testing would be needed to assess whether it is a result of our particular stimulus.
